# Self‐Adaptive Non‐Flammable Wallpaper With Layered Bead‐Network Structure for Light Path Modulation Enabling on‐Demand Building Thermal Management

**DOI:** 10.1002/advs.75716

**Published:** 2026-05-19

**Authors:** Jianyu Wu, Linmin Xia, Jiankun Wu, Yuying Yang, Wuhong Zhong, Jianguo Li, Yan Yu, Rilong Yang

**Affiliations:** ^1^ College of Material Engineering Fujian Agriculture and Forestry University Fuzhou China; ^2^ National Forestry and Grassland Administration Key Laboratory of Plant Fiber Functional Materials Fuzhou China; ^3^ School of Food and Liquor Engineering Sichuan University of Science and Engineering Yibin China

**Keywords:** building thermal management, hydroxyapatite nanowire, radiative cooling, solar heating, thermochromism

## Abstract

Self‐adaptive materials capable of radiative cooling and solar heating without extra energy consumption have drawn extensive interest. However, limited solar reflectivity in cooling mode and unsatisfactory spectral conversion ability, arising from color residue and weak scattering ability of adaptive color‐changing components, restrict their practical application. Herein, a layer‐by‐layer embedding strategy was proposed, in which thermochromic microcapsules were incorporated layer by layer into a layered network formed by inorganic hydroxyapatite nanowire bundles. The formed multi layered bead‐network structure achieved selective layer‐by‐layer reflection or absorption through light path regulation, overcoming the color residue limitation of thermochromic microcapsules. The obtained wallpaper exhibited a high reflectivity of 94.1% and an emissivity of 95.5% in cooling mode, along with a solar spectral regulation capability of 30.4%. This wallpaper had self‐adaptive ability to switch modes in response to temperature changes. In summer, the maximum cooling effect could reach 5°C. Conversely, in cold winter, it achieved a solar heating effect of 8.5°C. Moreover, outstanding flame‐retardant and self‐cleaning properties of wallpaper eliminated potential safety hazards, and prevented failure caused by contamination of rainwater and dust. This study presents a simple yet efficient approach to integrating contradictory optical properties, and provides a promising solution for building energy efficiency.

## Graphical abstract

A layered bead‐network wallpaper is designed to modulate the light propagation path, overcoming the color residue limitations of thermochromic components for efficient automatic switching between radiative cooling and solar heating modes. The layered bead‐network wallpaper provides passive, all‐season thermal regulation while offering self‐cleaning and flame‐retardant properties, presenting a sustainable solution for intelligent building energy management.

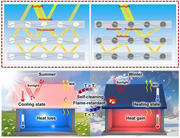

## 1 Introduction

Creating a comfortable living temperature is crucial for enhancing human well‐being, but it also leads to substantial energy consumption. This is particularly evident in the fact that air conditioning and heating systems account for 47% and 36% of total building energy use [1]. To reduce energy consumption and carbon emissions, the development of more eco‐friendly and energy‐efficient technologies for building thermal management has attracted growing interest.

Recent years, the daytime radiative cooling technology has emerged as a promising solution for energy‐efficient building cooling [2, 3, 4]. The daytime radiative cooling technology utilizes the 3K outer space as a cold sink, achieving sub‐ambient cooling by reducing solar heat absorption and maximizing heat dissipation via infrared thermal radiation to outer space [5]. The key factors for daytime radiative cooling materials are high reflectivity in the solar band (R_solar_) and emissivity within the atmospheric transparent window (E_ATW_). By tailoring micro‐nano scattering structures to enhance R_solar_ and selecting substrates with suitable chemical bond vibrations [6], many housing cladding materials with daytime radiative cooling function have been developed, including aerogels [7, 8], coatings [9, 10], films [11, 12], and wallpapers [13, 14]. To facilitate the practical application of radiative cooling cladding materials, various functional integration strategies have been extensively investigated. These include endowing materials with hydrophobic self‐cleaning properties to mitigate optical performance degradation caused by rainwater and dust contamination [15, 16], as well as introducing flame‐retardant functionality to eliminate potential safety hazards [17].

However, single‐mode radiative cooling materials can hardly meet the requirements of on‐demand building thermal management. During cold nights and in freezing weather, excessive cooling arising from passive radiation even exacerbates energy consumption for building heating [18]. To meet both cooling and heating demands, multi‐mode spectral regulation materials have been developed, primarily by modulating emissivity to control radiative cooling capacity or adjusting reflectivity to harvest energy from both outer space and the sun [19]. Various active regulation methods have been proposed, including electrochromic emissivity modulation of WO_3_ nanocrystals [20], flippable bilayer reflectivity [21], and deformation‐induced reflectivity changes in flexible films [22] and elastic aerogels [23]. Although these active systems exhibit regulatory capabilities, they require additional energy for mode switching, limiting their practical applicability.

Compared with active regulation, self‐adaptive regulation methods that aim to achieve autonomous mode switching through environmental stimuli are more sustainable and practical. To achieve autonomous switching between two contradictory optical properties, various environmentally responsive ingredients have been introduced, such as wettability‐induced thermo‐sensitive hydrogels [24], VO_2_ phase transition‐induced emissivity changes [25], humidity‐responsive materials [26], shape memory materials [27], reversible micro‐crack‐regulated emissivity [28], photochromic materials [29], and thermochromic materials [30]. Among them, self‐adaptive thermochromic materials have a natural advantage in on‐demand building thermal management owing to their direct correlation with temperature and adjustable transition temperature [31]. However, due to the inherent color residue and weak scattering ability of the thermochromic components, the R_solar_ of the self‐adaptive thermochromic materials can hardly meet the requirements for sub‐ambient cooling (R_solar_ ≥ 90%). Currently, a common approach to address this issue is to reduce the amount of thermochromic components added [30, 32]. Nevertheless, this practice compromises the spectral regulation capability of self‐adaptive thermochromic materials, resulting in unsatisfactory solar heating efficacy. There is a trade‐off between solar heating and sub‐ambient cooling. Therefore it remains a challenge for self‐adaptive thermochromic materials to simultaneously achieve enough R_solar_ in radiative cooling mode and satisfactory spectral regulation capabilities [33].

**FIGURE 1:**
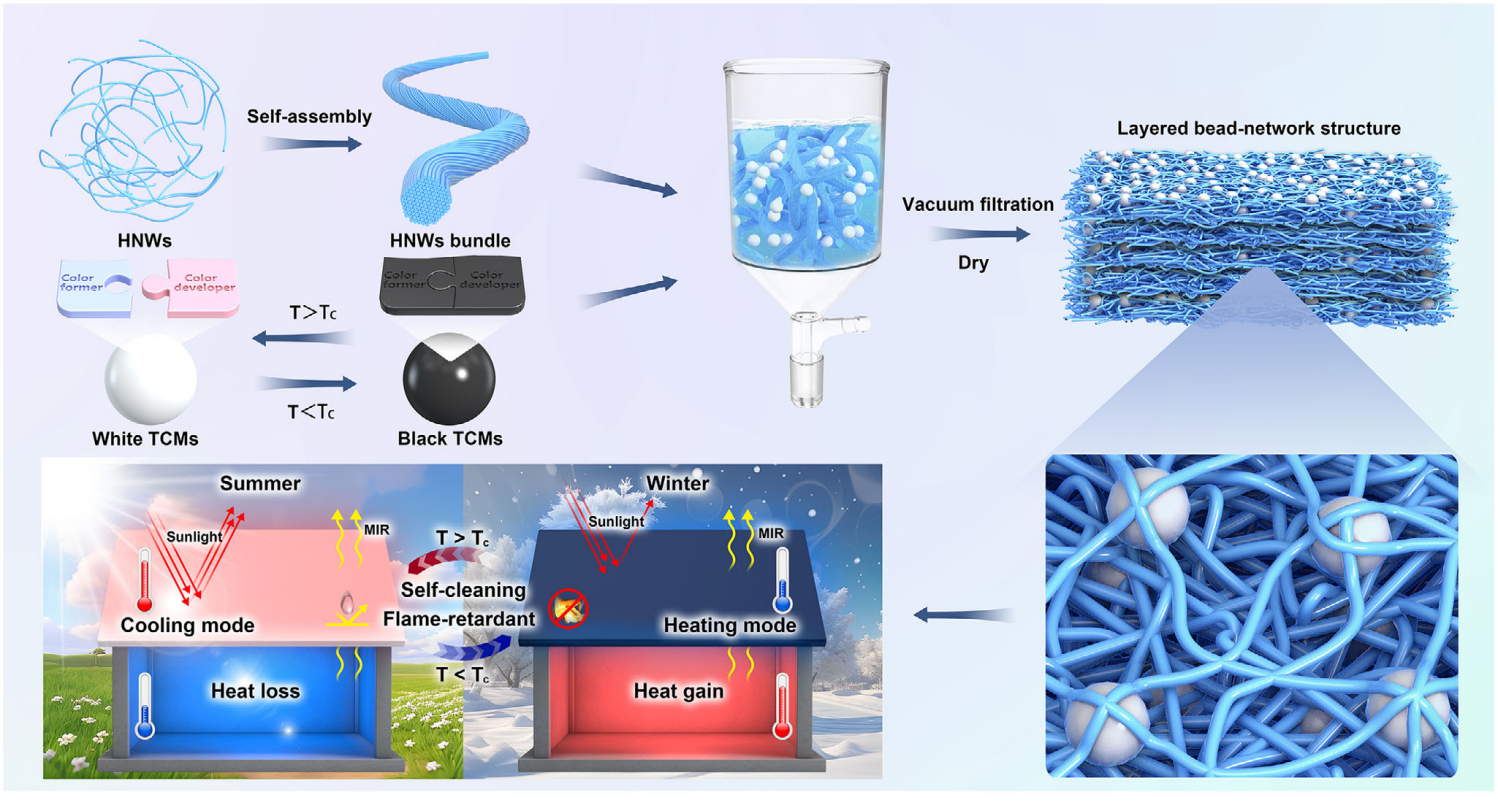
Schematic illustration for the multilayered bead‐network structure and the self‐adaptive building thermal management of the TCIP.

Herein, a thermochromic inorganic paper (TCIP) with excellent spectral regulation performance was prepared via a layer‐by‐layer embedding strategy, which can autonomously switch between radiative cooling and solar heating based on ambient temperature (Figure 1). In this strategy, self‐assembled hydroxyapatite nanowires (HNWs) bundles exhibit strong broadband Mie scattering capability. These bundles interweave and stack into a multi‐layered network, and thermochromic microcapsules (TCMs) are incorporated layer by layer into the layered network, thus forming a multi‐layered bead‐network structure. This structure achieves selective layer‐by‐layer reflection or absorption by regulating the propagation path of light, overcoming the color residue limitation of thermochromic microcapsules (TCMs), thus endowing TCIP with effective adaptive thermal management capability. TCIP appears white in cooling mode (R_solar_ = 94.1%) and dark gray in heating mode (R_solar_ = 63.7%), achieving 30.4% spectral regulation capability. Combined with its 95.5% emissivity in the atmospheric window, it realizes effective thermal regulation: a maximum 5°C cooling effect in summer and an 8.5°C solar heating effect in winter. Moreover, TCIP's high‐roughness bead‐network structure enables easy achievement of high hydrophobicity (a water contact angle of 147°). Additionally, the inorganic nature of HNWs endows TCIP with inherent flame retardancy. TCIP can serve as a green and safe building cladding material, and holds broad prospects in terms of building energy efficiency and thermal management.

## 2 Results and Discussion

### 2.1 Design Principles, Preparation and Bead‐Network Structure of TCIP

**FIGURE 2:**
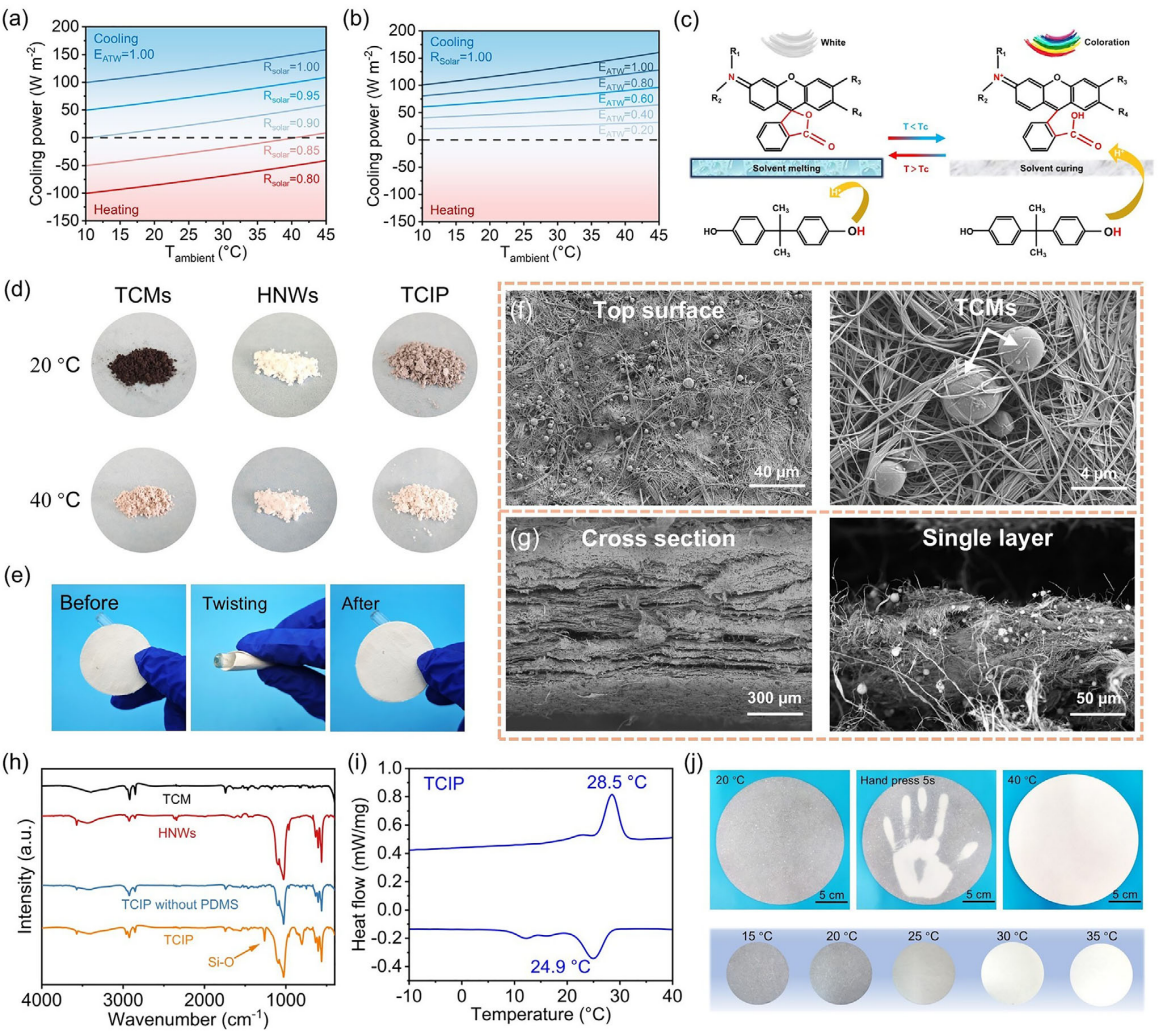
(a) Cooling power at fixed emissivity (E_ATW_ = 1) under varying solar reflectivity. (b) Cooling power at fixed reflectivity (R_solar_ = 1) under varying emissivity. (c) Schematic diagram illustrating the thermochromic mechanism of TCMs. (d) Digital photographs of the thermochromic dry pulp and demonstration of its color‐changing effect. (e) Digital image showing the flexibility of TCIP. (f) The SEM image of the TCIP top surface. (g) The SEM images of the TCIP cross‐section and the morphology of its single layer. (h) The FTIR spectrum of samples. (i) The DSC curve of TCIP. (j) Digital images illustrating TCIP's rapid thermochromic reaction and its dynamic color transitions with temperature variations.

The thermal equilibrium directionality of the self‐adaptive radiative cooling materials is determined by the net cooling power. When the temperature of a radiative cooling material equals the ambient temperature, the positive or negative value of the net cooling power directly dictates whether the final equilibrium temperature is lower or higher than the ambient temperature. The net cooling power of radiative cooling materials is expressed by the equation: P_cool_ = P_rad_−P_amb_−P_sun_−P_con+cov_ [34]. Here, P_cool_ denotes the net cooling power, P_rad_ represents the heat radiated from the material surface to outer space, P_amb_ is the heat radiated from the environment to the material surface, P_sun_ is the solar heat absorbed by the material surface, and P_con+cov_ signifies the heat exchanged via convection and conduction. The specific calculation process is provided in Note S1. With E_ATW_ fixed at 1, the net cooling power under different R_solar_ conditions is shown in Figure 2a. To achieve the net cooling power >0, at least 90% of sunlight must be reflected. If R_solar_ is below 90%, the material switches from cooling to heating. In addition, with R_solar_ fixed at 1, the net cooling power under different E_ATW_ conditions is shown in Figure 2b. Compared with the significant impact of reflectivity on net cooling power, the influence of E_ATW_ is relatively minor. For instance, when R_solar_ decreases to 80%, the net cooling power drops sharply. In contrast, as E_ATW_ decreases, the net cooling power only declines slightly. The results of the theoretical calculations indicate that R_solar_ regulation is a more efficient way to achieve self‐adaptive building thermal management than emissivity regulation, and an R_solar_ greater than 90% in cooling mode is an essential requirement for self‐adaptive building thermal management materials to achieve sub‐ambient cooling. Additionally, to achieve satisfactory cooling and heating mode switching, self‐adaptive building thermal management materials need to have high spectral regulation capability.

To achieve self‐adaptive building thermal management, thermochromic microcapsules (TCMs) are used as the adaptive functional component. As shown in Figure 2c, TCMs consist of a shell and a thermochromic three‐component system (color former, color developer, solvent). The solvent's melting point equals the thermochromic temperature, enabling on‐demand preparation of TCMs with varied transition temperatures. Below the melting point, solid solvent allows the color developer (bisphenol A) to protonate the color former (fluorane dye), opening its lactone ring into a conjugated π‐system that absorbs visible light and appears colored. Above the melting point, melted solvent disrupts their interaction, and the color former regains electrons. The conjugated system is broken, allowing it to reflect sunlight and revert to white. Their tunable transition temperature and distinct color change have enabled the application of TCMs in adaptive thermal management materials. However, the incorporation of TCMs is commonly associated with the issue of color residue. As shown in Figure S1a, in the white mode above the transition temperature, the color residue of the TCMs reduces R_solar_, resulting in the failure of daytime radiative cooling. Meanwhile, the scattering ability of micron‐scale TCMs alone is insufficient to achieve broad‐spectrum high reflectivity. To achieve adequate R_solar_, the amount of TCMs incorporated needs to be reduced. However, this compromises the spectral regulation capability of self‐adaptive thermochromic materials, leading to poor solar heating efficacy. In addition, the poor interfacial bonding between TCMs exacerbates the difficulty of constructing micro‐nano structures to realize broad‐spectrum high reflectivity (Figure S1b).

To overcome the trade‐off between R_solar_ in cooling mode and spectral regulation capability, a strategy of light propagation path regulation is proposed by selecting materials with specific optical properties and designing scattering structures. The main considerations are as follows: 1. Hydroxyapatite nanowires (HNWs) with extremely low extinction coefficients are selected as the basic framework. The light absorption of HNWs is very low, leaving only reflection or transmission. 2. For diffuse scatterers, reflection is the overall result of multiple scattering of light within the material. HNWs can self‐assemble into bundles, and their adapted diameter distribution generates strong Mie scattering. The multi‐layered HNWs bundle network ensures overall high reflectivity by controlling layer‐by‐layer backscattering of light (Figures S2 and S3). 3. The added TCMs and HNWs bundles form a layered bead‐network structure, and the nanowire bundle network can firmly bind the micron‐scale TCMs, resulting in good adhesion between them. 4. TCMs not only introduce dual‐mode reflectivity, but their inherent micron size is utilized to reflect the near‐infrared wavelength band. 5. The selected TCMs exhibit different light absorption capacities depending on the ambient temperature. In the low‐absorption white state, the strong backscattering capability of the HNWs framework enables a large amount of light to be reflected rapidly, which reduces the overlapping absorption between TCMs, alleviates the defect of color residue, and thus maintains high reflectivity. In the high‐absorption black state, the light absorption capacity of TCMs far exceeds that of the HNWs bundle framework. Photons are captured by TCMs during multiple scattering within the HNWs bundle framework, ensuring high spectral regulation capability.

Based on this design principle, a thermochromic inorganic wallpaper (TCIP) with a multi‐layered bead network structure has been developed for on‐demand building thermal management. The thermochromic inorganic wallpaper can be easily prepared via a simple pulping and papermaking process, and the forming process does not involve toxic chemical reagents. The transition temperature of the TCMs was selected as 28°C, primarily based on considerations of the human thermal comfort range [35]. As shown in Figure 2d, homogeneously mixing HNWs with TCMs yields thermochromic dry pulp, which facilitates storage and transportation. Owing to the introduction of black thermochromic microcapsules with a transition temperature of 28°C, the dry pulp exhibited distinct temperature‐dependent color change behavior: it appeared gray at 20°C to absorb sunlight and turned white at 40°C to reflect sunlight. As shown in Figure S4, the dry pulp was mixed with water and homogenized to obtain wet pulp, and TCIP was formed via a vacuum‐assisted filtration and drying process. The TCIP can be further immersed in a PDMS solution to obtain high hydrophobicity. The TCIP demonstrates excellent flexibility (Figure 2e) and mechanical properties (Figure S5 and Movie S1), which can be arbitrarily twisted into desired shapes and withstand 3200 times its own weight without breaking. The convenient fabrication process of TCIP endows it with scalability to address diverse application scenarios. When high mechanical strength is required, TCIP can be further reinforced by incorporating a small amount of nanocellulose, thereby achieving a high tensile strength of up to 37 MPa, though at the cost of some reduction in reflectivity (Figure S6). This is because nanocellulose facilitates the formation of a denser and mechanically stronger structure; however, the concomitant reduction in porosity leads to a decrease in the number of available scattering interfaces. In addition, the inherent inorganic bundled network of TCIP confers high flame‐retardant performance.

The surface SEM image of TCIP showed that TCMs were uniformly distributed within the HNWs bundle network (Figure 2f). A higher‐magnification image revealed that TCMs and HNWs collectively formed a bead‐network structure, with TCMs tightly bound in the HNWs bundle network. The cross‐sectional SEM image of TCIP (Figure 2g) exhibited a multi‐layered structure, with TCMs evenly distributed in each layer, achieving uniform layer‐by‐layer dispersion of TCMs. The TCMs were firmly embedded within the HNWs bundle network, preventing their detachment under external forces (Figure S1c). The FTIR spectrum of TCIP displayed overlapping peaks from both HNWs and TCMs, confirming successful composite formation (Figure 2h). After PDMS modification, a distinct peak attributed to the Si‐C bond appeared at 800 cm^−^^1^ in the FTIR spectrum. Si element mapping in EDS further confirmed the successful modification of PDMS (Figure S7). The DSC characterization of TCIP showed a sharp and precise exothermic peak at 28.5°C (Figure 2i), consistent with the exothermic peak of TCMs (Figure S8). This indicates that TCIP possesses thermochromic transition temperature consistent with TCMs. At 20°C, TCIP exhibited a distinct gray color, and it turned white rapidly 2 seconds after being pressed with the hand (Figure 2j and Movie S2). The color of TCIP varied with temperature, enabling rapid adaptation to real‐time weather conditions.

### 2.2 Dual‐Mode Optical Properties and Light Path Regulation Design of TCIP

**FIGURE 3:**
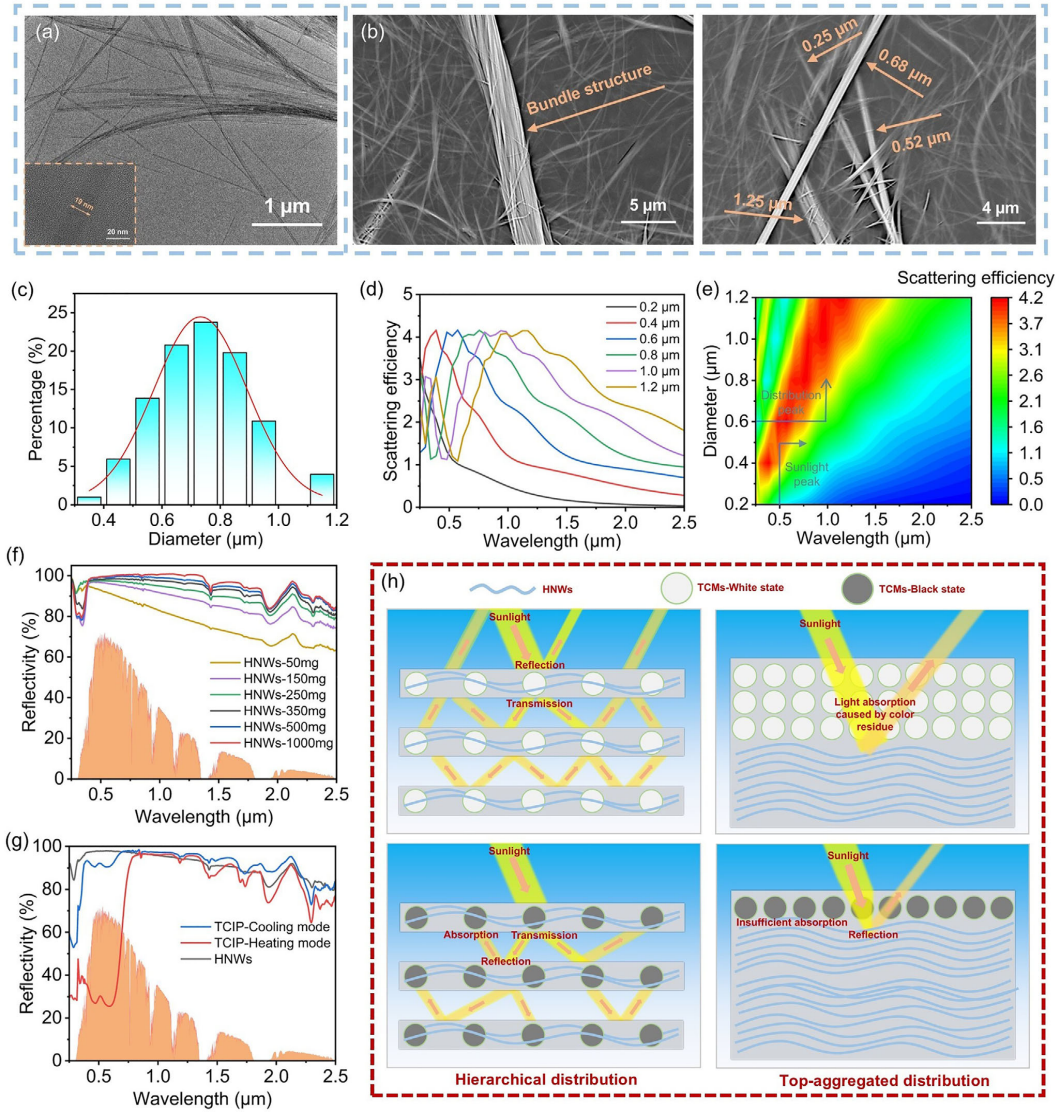
(a) The TEM images of HNWs. (b) The SEM images of HNWs bundles with different diameters. (c) Diameter distribution of HNWs bundles. (d) The FDTD‐simulated scattering efficiency of HNWs with different diameters in the solar wavelength range. (e) Scatter efficiency heat map of HNWs with different diameters in the solar wavelength range. (f) Solar reflectance spectra of HNWs with varying solid contents. (g) The reflectivity spectra of TCIP in both cooling and heating modes, as well as HNWs paper. (h) Schematic diagram of light propagation paths of the two TCMs distribution: hierarchical distribution (left) and top‐aggregated distribution (right).

According to Mie scattering theory, when the diameter of the scatterer is close to the wavelength of the incident light, a strong scattering effect is generated [36]. The self‐assembly behavior of HNWs plays a critical role in the high reflectivity of the TCIP. The prepared HNWs slurry appeared pale yellow and turned white after cleaning and self‐assembly (Figure S9). In the TEM images of the HNWs slurry, there was steric hindrance between HNWs due to the presence of residual oleic acid (Figure 3a). Individual HNWs possessed an ultra‐high aspect ratio (with nanoscale diameter and length of tens of micrometers) and exhibited single‐crystal diffraction patterns. After removing oleic acid with ethanol and deionized water, HNWs self‐assembled into nanowire bundles of varying diameters due to the elimination of steric hindrance and the presence of abundant surface functional groups. HNWs were aggregated into bundles with a wide diameter distribution, and exhibited good flexibility (Figure 3b). The diameter distribution of HNWs bundles ranged from 200 nm to 1.2 µm, with the highest frequency in the 600–700 nm range (Figure 3c). Compared to the pristine HNWs slurry, the self‐assembled HNWs bundle pulp exhibited much stronger light scattering effect and less light transmission when they were dispersed in water (Figure S10).

Another key factor is the refractive index difference between adjacent media. The degree of light deflection at the interfaces increases with the difference in their refractive index [37]. HNWs had a refractive index of 1.4 and near‐zero extinction coefficient (Figure S11). This indicates that light incident from air (refractive index of air is 1) undergoes significant deflection at their interface. The extremely low extinction coefficient means that HNWs barely absorb light [38]. Finite‐difference time‐domain simulations were carried out to investigate the light‐scattering capacities of HNWs bundles with different diameters (Figure S12 and Note S2). The simulation results showed that HNWs bundles with different diameters exhibited varying light‐scattering capacities, and they exhibited different scattering effects on light of different wavelengths (Figure 3d). Aligned with Mie theory, HNWs bundles efficiently scattered light wavelengths comparable to their diameter. The peak HNWs diameter distribution corresponded to the solar irradiance peak, laying the foundation for broadband high solar reflectivity (Figure 3e).

Increasing the number of layers enhanced backward scattering probability, boosting overall reflectivity. The number of layers inside the HNWs papers was determined by the solid content of HNWs, where the solid content equals the dry weight of HNWs and does not include any other materials. Therefore, the solid content was also an important parameter that affected the light reflectivity. The R_solar_ of HNWs papers with different solid contents was calculated using the method described in Note S3. Figure 3f showed the R_solar_ of HNWs papers with different solid contents: 86.4% (50 mg), 92.7% (150 mg), 96.1% (250 mg), 97.0% (350 mg), 98.1% (500 mg), and 99.2% (1000 mg). This trend is attributed to multiple Mie scatterings occurring as light passes through the multi‐layered nanowire bundles network. With a negligible extinction coefficient, insufficient layers lead to transmission rather than absorption (Figure S13). At 1000 mg solid content, nearly all light is reflected (>99%). However, considering the addition of TCMs and the decrease in paper flexibility when the solid content is too high, the solid content of HNWs was set at 250 mg in subsequent experiments. By selecting hydroxyapatite nanowires with a high refractive index and regulating their self‐assembly behavior and micro‐layered structure, an inorganic network framework with high solar reflectivity can be constructed. The high reflectivity enabled by the laminated structure with efficient Mie scattering lays the foundation for TCIP's high cooling‐mode reflectivity.

To achieve self‐switching between radiative cooling and solar heating, thermochromic microcapsules (TCMs) were introduced. The thicknesses of TCIP with 0, 25, 50, and 100 mg TCMs are 0.555, 0.617, 0.663, and 0.761 mm, respectively. The reflectance spectra of TCIP with different TCMs in two modes were shown in Figure S14a and b. When the addition amount of TCMs exceeded 50 mg, there was no significant change in the solar reflectivity of the TCIP in heating mode. Therefore, the addition amount of TCMs was set at 50 mg. Compared with pure HNWs, TCIP showed an increased reflectivity in the near‐infrared band (1.0–2.5 µm) (Figure 3g). This is attributed to the 1–5 µm diameter distribution of TCMs (Figure S15). The micron‐sized TCMs enhance light reflection in this wavelength band. The R_1.0–2.5 µm_ range for TCIP was 94.6%, which presented a 2% increase compared to that of HNWs paper (92.6% in the same 1.0–2.5 µm range). Meanwhile, color residue of TCMs in the visible range (Figure S1) is significantly reduced, enabling TCIP to exhibit 94.1% high R_solar_ in the cooling mode. This result indicates that the addition of TCMs introduces heating mode without compromising cooling reflectivity (Figure 3g). The TCIP had a reflectivity of 63.7% in the heating mode, approaching the light absorption limit of pure TCMs powder (Figure S1). This indicates that the light‐absorbing capacity of TCMs is well maintained in TCIP. Eliminating color residue and achieving satisfactory light absorption endow TCIP with a solar spectral regulation capacity up to 30.4%, enabling efficient on‐demand building thermal management.

Besides the high scattering efficiency of nano‐scale HNWs and micro‐scale TCMs, the light path modulation of multi‐layered bead‐network structure plays a significant role in high spectral regulation capability (Figure 3h). In the cooling mode, TCMs in the white state exhibit low light absorption. However, the presence of the color former endows them with certain light‐absorbing behavior, rendering them an imperfect sub‐reflector. When the TCMs are aggregated within the materials, the light absorption generated by the TCMs is accumulated and enhanced, thereby causing the phenomenon of color residue. Taking the top‐aggregated distribution as an example, multiple reflection and absorption of incident light occurs between aggregated white‐state TCMs, resulting in the increase of the light absorption. Compared with the top‐aggregated distribution, the hierarchical distribution of the TCMs due to the multilayered bead‐network structure has obvious advantages in terms of light propagation. Hierarchical distribution ensures monodispersion of TCMs within HNWs bundles, preventing TCMs aggregation and light absorption stacking. This allows most light to escape via scattering or reflection.

In the heating mode, the black‐state TCMs exhibit high light absorption. In the top‐aggregated distribution, to achieve a reflectivity of over 90% in the cooling mode, it requires limiting the addition amount of TCMs. This leads to insufficient light absorption in the heating mode. In the hierarchical distribution, the scattering interface of the multi‐layered HNWs bundle network acts as an aid for light absorption. The TCMs with high light absorption act as light traps. Once photons encounter them during propagation, they are absorbed. The interaction between the scattering interfaces of HNWs bundles and TCMs light traps endows TCIP with high light absorption capacity.

**FIGURE 4:**
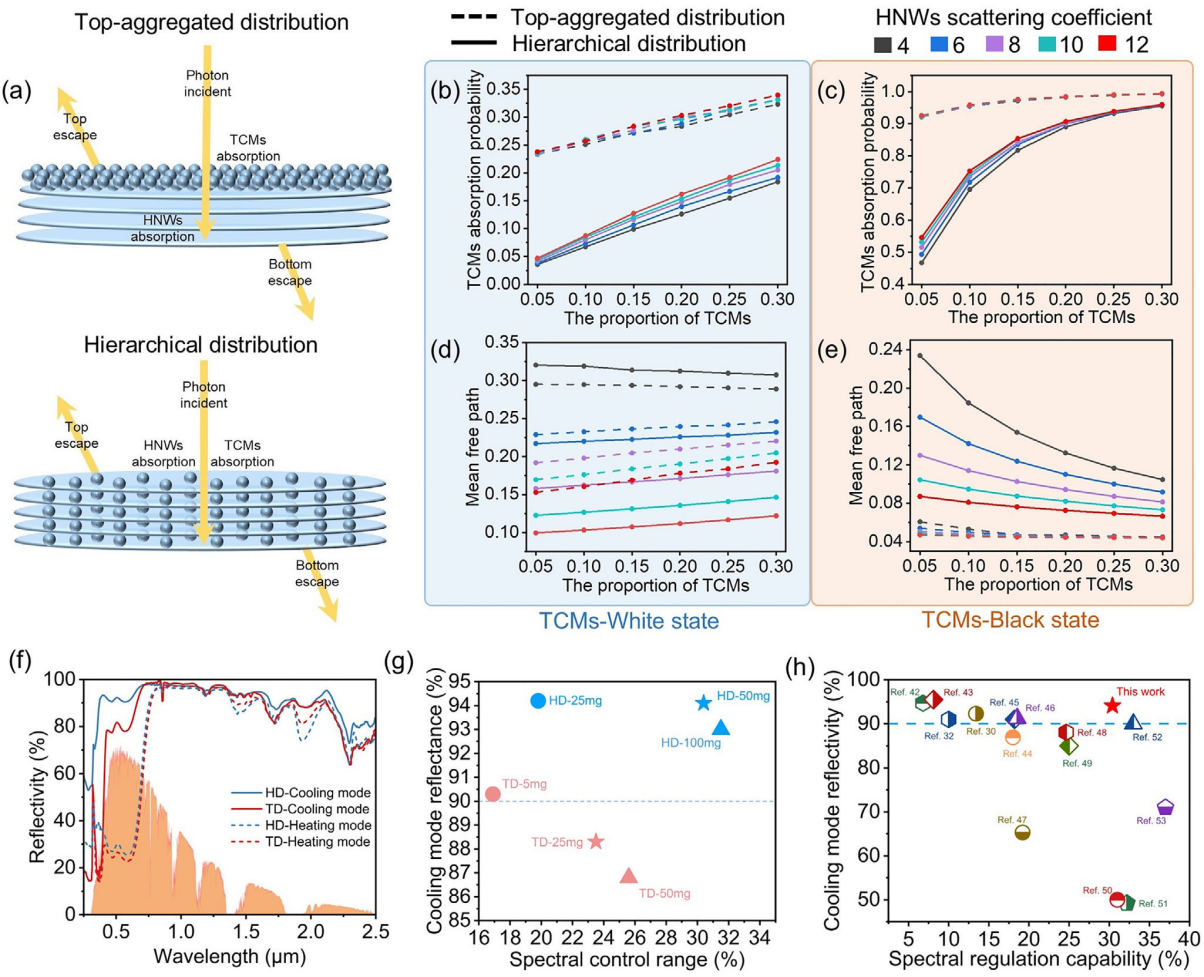
(a) Schematic diagram of the Monte Carlo photon transport simulation model comparing the light propagation paths of hierarchical distribution and top‐aggregated distribution. (b–e) The TCM absorption probabilities and mean free paths obtained from Monte Carlo photon transport simulations under different TCM contents and HNWs scattering coefficients: (b) TCMs absorption probability in the TCMs‐white state. (c) TCMs absorption probability in the TCMs‐black state. (d) Mean free paths in the TCMs‐white state. (e) Mean free paths in the TCMs‐black state. (f) The reflectance spectra of the hierarchical distribution (HD) and the top‐aggregated distribution (TD). (g) Contrast diagrams comparing cooling mode reflectance and spectral tuning capability between HD and TD. (h) Contrast diagrams comparing cooling mode reflectance and spectral tuning capability between TCIP and other TCM‐based thermal management materials [30, 32, 42, 43, 44, 45, 46, 47, 48, 49, 50, 51, 52, 53].

To verify that the layered bead‐network structure overcomes the color residue limitation of TCMs through light path modulation, Monte Carlo photon transport simulations were performed [39, 40, 41]. The radiative transfer equation is a fundamental differential‐integral equation that describes the transfer of energy as a result of absorption, scattering, and other effects when light propagates through a participating medium. The Monte Carlo method was employed to solve the radiative transfer equation by simulating the random walk of a large number of photons within a multilayered HNWs/TCMs composite model. The absorption and mean free path of photons were then statistically analyzed. The simulation adopted a plane‐parallel slab geometry. The slab is infinite in the lateral directions and has a finite thickness in the z‐direction. It was uniformly divided into 20 layers along the thickness direction (Figure 4a). The two distribution models were distinguished by the spatial arrangement of TCMs. In the hierarchical distribution, TCMs were uniformly distributed across all layers. In the top‐aggregated distribution, TCMs preferentially populated the top layers with the total TCM content strictly equal to that of the hierarchical distribution. The detailed simulation process is provided in Note S4 and Table S1.

Different TCMs contents and HNWs scattering coefficients were investigated to distinguish the dominant mechanism that suppresses color residue. The simulation results are presented in Figure 4b–e. When TCMs were in the white state (imperfect sub‐reflector), the TCMs absorption probability for the hierarchical distribution was consistently significantly lower than that for the top‐aggregated distribution. This indicates that the spatial separation of TCMs in the hierarchical distribution effectively reduces the absorption stacking effect among TCMs in the low absorption state, thereby suppressing color residue. Furthermore, compared with the top‐aggregated distribution, the TCMs absorption probability in the hierarchical distribution was more sensitive to variations in the HNWs scattering coefficient. This observation was consistent with the trend in mean free path. The hierarchical distribution exhibited a smaller mean free path, suggesting a higher frequency of scattering or absorption events for photons within this structure. When TCMs were in the black state (light trapping), the TCMs absorption probabilities for the two distributions gradually converged as the TCMs content increased. The smaller mean free path observed in the top‐aggregated distribution was attributed to the immediate high‐probability absorption of photons upon entering the TCMs‐enriched top layer, resulting in a significantly shortened transport distance. For the hierarchical distribution, the mean free path gradually decreased with increasing HNWs scattering coefficient. This indicates that the enhanced scattering capability of the HNWs framework increased the frequency of photon–medium interactions. The increased frequency of scattering events enhances the interaction between photons and the TCM light traps, thereby increasing the TCMs absorption probability. For instance, at a TCMs volume fraction of 0.25 in the white state, the absorption probability of the hierarchical distribution was 13.2% lower than that of the top‐aggregated distribution. At the same TCMs volume fraction of 0.25 in the black state, the absorption probability of the hierarchical distribution was only 5.2% lower than that of the top‐aggregated distribution.

In summary, the Monte Carlo simulations quantitatively revealed the regulatory mechanisms of light propagation paths for the two TCMs distribution structures. In the white state, hierarchical distribution effectively suppressed the absorption stacking effect through spatial separation of TCMs, significantly reducing the TCMs absorption probability and increasing the mean free path, thereby mitigating color residue. In the black state, the high absorption coefficient of TCMs converts them into efficient light traps. In the hierarchical distribution, the strongly scattering HNWs framework ensures effective photon capture, resulting in a TCMs absorption probability comparable to that of the top‐aggregated distribution. Therefore, the hierarchical distribution maintains effective photothermal conversion in the heating mode. The hierarchical distribution is more favorable for enhancing the cooling‐mode reflectance and overall spectral modulation capability.

The measured reflectance spectra of the two distributions at different TCM addition amounts are shown in Figure S14. When both distributions have a TCM addition amount of 50 mg, the hierarchical distribution exhibited higher cooling‐mode reflectivity. Moreover, the hierarchical distribution does not show a significant decrease in absorbance in the heating mode because of the interaction between HNWs bundles' scattering interfaces and TCMs light traps (Figure 4f). The comparison chart of different TCMs addition amounts under the two distributions is shown in Figure 4g. When 5 mg of TCMs is added, the top‐aggregated distribution only achieves a reflectivity of 90% (with 17% spectral modulation). Additionally, a further increase in TCMs significantly reduces the cooling‐mode reflectivity. In contrast, due to its stronger scattering capability and light path control, the hierarchical distribution formed by the layered bead‐network structure balances the cooling reflectivity and spectral modulation. When the amount of TCMs is 50 mg, its cooling reflectivity reaches 94.1%, and the spectral modulation is 30.4%. The consistency between the simulation results and experimental observations confirms the underlying mechanism by which the layered bead‐network structure achieves the synergistic optimization of high reflectivity in the cooling mode and spectral regulation capability, providing support for the light path modulation strategy. Due to these structural advantages, TCIP outperforms most TCMs‐based on‐demand thermal management materials in terms of cooling reflectivity and spectral modulation (Figure 4h and Table S2).

### 2.3 Radiative Cooling Performance and Solar Heating Performance of TCIP

**FIGURE 5:**
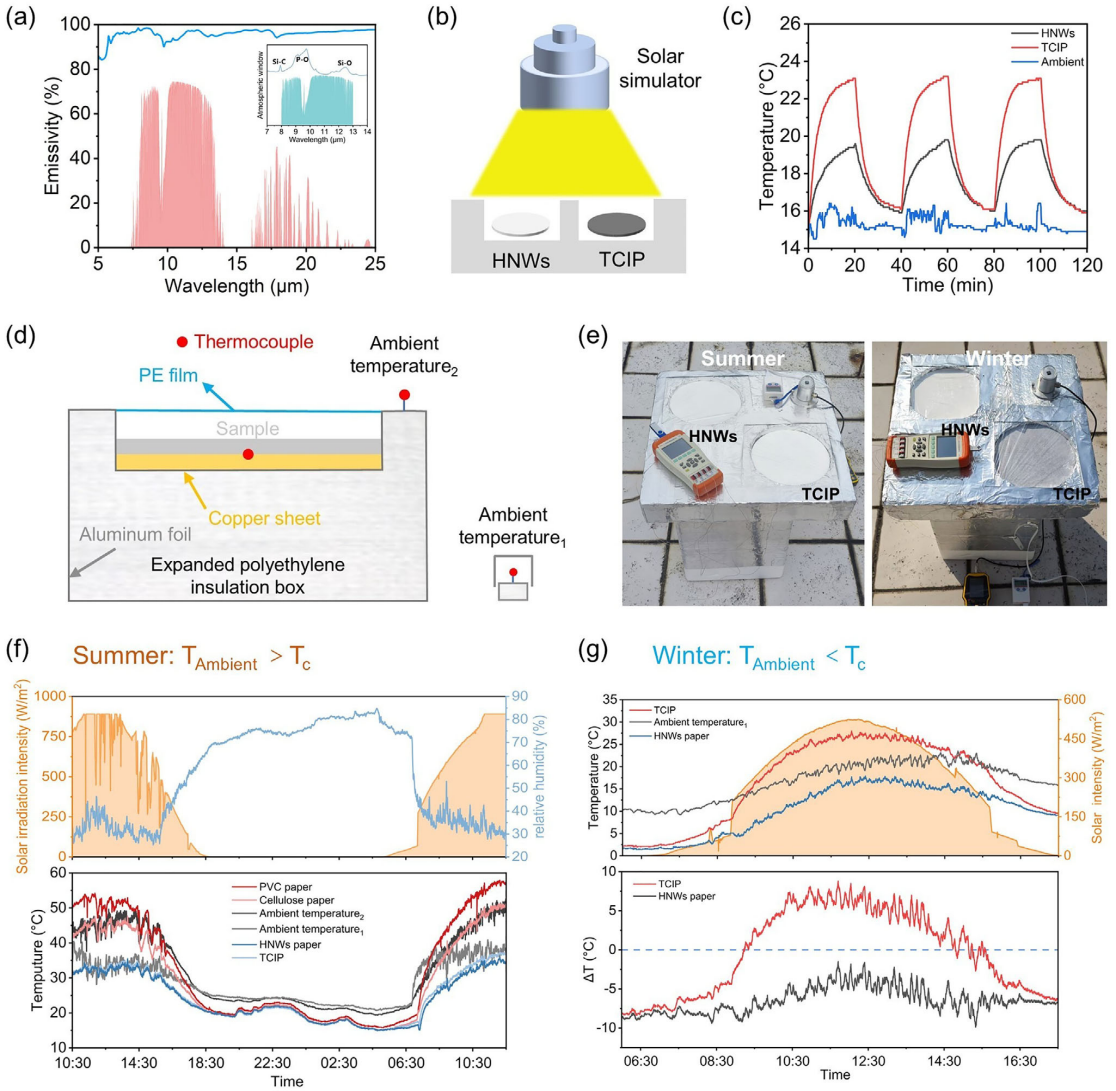
(a) Emissivity spectrum of TCIP. (b) Schematic diagram of the xenon lamp solar simulator used for indoor solar absorption tests. (c) Temperature variation curves of TCIP and pure HNWs paper under xenon lamp. (d) Schematic diagram of the outdoor temperature testing device. (e) Digital images of TCIP in summer (white, cooling mode) and winter (gray, heating mode). (f) Summer outdoor temperature variation curves of TCIP, HNWs paper, commercial PVC paper, and cellulose paper. (g) Winter outdoor temperature variation curves of the TCIP and HNWs paper.

Besides solar reflectivity, E_ATW_ is also critical for efficient cooling. The emissivity of the atmospheric window determines the heat exchange capability between thermal management materials and outer space, and it constitutes the foundation for achieving radiative cooling. TCIP exhibited a high E_ATW_ of 95.5% in the atmospheric window (Figure 5a) due to the vibrational absorption of abundant phosphate groups in HNWs that match the atmospheric window. Additionally, the modification of PDMS introduces Si‐C and Si─O bonds [54], which also lie within the atmospheric window, further enhancing the E_ATW_.

To verify the solar absorption performance of TCIP, simulated solar irradiation experiments were conducted under indoor natural convection. A xenon lamp with an AM 1.5 optical filter was used as the solar simulator (Figure 5b), and pure HNWs paper was selected as the control. Since there was no outer space cold source indoors, temperature changes depended solely on heat generated by photothermal conversion and heat exchange with indoor air. The solar irradiance incident on the sample was maintained at 1000 W/m^2^. The indoor environment was approximately 15 °C. The light was turned on and off for 20 minutes each, and this process was repeated three times. As shown in Figure 5c, TCIP remained in heating mode because the indoor temperature was below the thermochromic temperature (28°C), while pure HNWs stayed white due to their single spectral mode. Upon simulative solar irradiation, the temperature of TCIP rose rapidly, which was due to the low reflectivity of TCIP in heating mode. The surface temperature of the TCIP was approximately 4°C higher than that of HNWs paper after 10 minutes, demonstrating effective solar heating at low temperatures. When the heat generated by photothermal conversion was balanced with the heat exchange between the indoor air and the TCIP, the surface temperature of the TCIP stabilized at 23°C. After turning off the xenon lamp, the temperatures of both samples dropped quickly and eventually converged, with consistent results across three cycles. To evaluate practical temperature management capability, outdoor tests were conducted using a homemade temperature measurement device (Figure 5d), consisting of an insulated box (aluminum foil‐covered exterior to reflect sunlight), a thermocouple thermometer, a light power meter, and a humidity meter. HNWs paper, commercial PVC paper, and cellulose paper served as controls. HNWs, PVC paper, and cellulose paper had reflectivities of 96.1%, 73.6%, and 77.3%, respectively (Figure S16). Samples were placed on copper sheets with thermocouples inside the box, covered with a transparent polyethylene film to eliminate air convection interference. Two ambient temperatures were monitored: (1) Ambient temperature_1_, defined as the air temperature unaffected by solar irradiation, which is a widely recognized reference temperature for evaluating radiative cooling performance. (2) Ambient temperature_2_, defined as the air temperature inside a sealed cavity under direct solar irradiation, which is exposed to the same solar exposure conditions as the sample. Previous studies have reported that the arbitrary use of these two temperatures as the ambient temperature has caused considerable confusion [55]. In this study, employing both ambient temperatures aims to enhance the reliability and comparability of field tests, thereby better elucidating radiative cooling performance under complex outdoor environments. Solar irradiance and humidity were also monitored in real time. Digital images of the samples showed that TCIP exhibited a cooling mode (white, with high reflectivity) in hot summers and a heating mode (gray, with low reflectivity) in cold winters (Figure 5e).

The summer test results were shown in Figure 5f. Even when the relative humidity reached 70%–80% at night, the TCIP could still achieve a cooling effect of 5.6°C. This effect is enabled by the high E_ATW_ of the TCIP, which transfers heat to outer space through the atmospheric window. The three controls also showed cooling effects due to their high atmospheric window emissivity [56, 57]. During daytime with 875 W/m^2^ solar irradiance and relative humidity ranging from 30% to 40%, TCIP remained in cooling mode, with a maximum temperature difference of 5°C lower relative to ambient temperature_1_. The HNWs paper had a slightly higher reflectivity, and its temperature dropped by 7°C. In contrast, the temperature of PVC paper reached 55 °C, and that of cellulose paper reached 47.5°C. These temperatures were 15°C and 7°C higher than ambient temperature_1_, respectively, and even higher than ambient temperature_2_. This is because PVC paper and cellulose paper have relatively low reflectivity. Both absorb excessive solar energy, causing their temperatures to exceed the ambient temperature in sunlight. PVC paper and cellulose paper have no daytime radiative cooling effect. The winter test results were shown in Figure 5g. Under daytime solar irradiation, TCIP operated in heating mode, and could effectively absorb solar light and convert the light energy into heat energy. The maximum solar irradiance in winter was 500 W/m^2^, and the daytime relative humidity was 40% (Figure S17). TCIP reached 8.5°C above ambient temperature_1_ at peak solar irradiance, stabilizing at a comfortable temperature of 28°C. HNWs paper, with constant high reflectivity, remained 2.4°C below ambient temperature_1_ and 10.9°C lower than TCIP, indicating that the single cooling mode causes excessive cooling in cold winters. These results suggest that TCIP exhibits adaptive building thermal management performance, which is conducive to reducing the building's cooling and heating energy consumption and improving indoor thermal comfort.

### 2.4 Self‐Cleaning Performance and Flame Retardant Performance of TCIP

**FIGURE 6:**
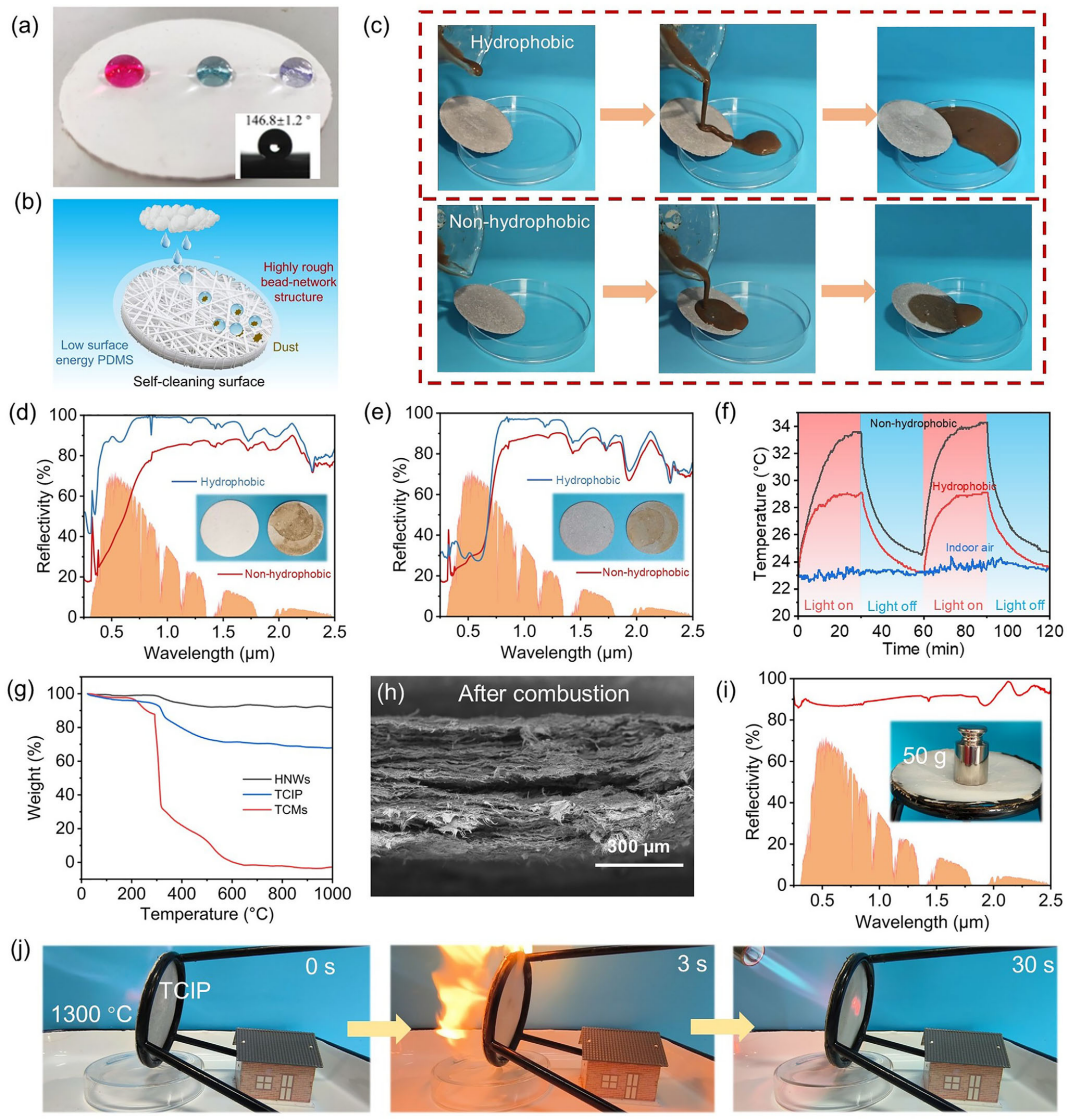
(a) Water contact angle measurement and digital image of TCIP. (b) Schematic diagram of TCIP's self‐cleaning property. (c) Digital photos demonstrate the mud pouring experiment on hydrophobic and non‐hydrophobic TCIP surfaces. (d, e) Digital photos of hydrophobic and non‐hydrophobic TCIP after the mud pouring experiment, along with their reflectance spectra: d) cooling mode and e) heating mode. (f) Temperature variation graphs of hydrophobic and non‐hydrophobic TCIP during indoor simulated solar irradiation tests. (g) Thermogravimetric curves of TCIP, TCMs, and HNWs. (h) SEM images of the TCIP cross‐section after flame exposure. (i) Reflectance spectra of TCIP after combustion and digital photos showing its ability to support a 50 g weight after combustion. (j) Demonstration of the flame‐blocking performance of TCIP.

In complex outdoor environments, the surface optical properties of radiative cooling materials are easily affected by moisture and dust [58]. The construction of hydrophobic surfaces can reduce moisture retention and dust adsorption, thereby maintaining their surface optical properties under complex conditions like dust pollution and preserving efficient cooling effects [59]. TCIP not only exhibits excellent thermal management performance but also high hydrophobicity. The water contact angles of samples with different TCMs loadings are presented in Figure S18. Although a superhydrophobicity can be achieved at higher TCMs loadings, the TCIP with a TCMs loading of 50 mg was ultimately selected based on the consideration of optical performance. As shown in Figure 6a, the water contact angle of TCIP was 146.8 ± 1.2°. The state of water droplets in contact with the TCIP surface was observed. The water droplets did not adhere to the TCIP surface and could still fall off completely (Figure S19). Water droplets could roll easily from the TCIP surface when TCIP was placed on a 2 mm‐thick wood chip with an inclination angle of 4° (Figure S20). TCIP also exhibited good mechanical wear resistance. The water contact angle of TCIP showed no significant change after 50 tape peeling tests and 25 sandpaper grinding tests (Figure S21). These results indicate that TCIP exhibits excellent hydrophobic properties, which are attributed to the micro‐nano rough surface formed by the bead‐network structure and the low surface energy of PDMS. Notably, the PDMS modification improved the hydrophobicity while preserving the thermochromic response kinetics, optical properties, and flame‐retardant performance of the HNWs/TCMs bead‐network structure (Table S3 and Figures S22–S25). This outstanding surface hydrophobicity endows TCIP with self‐cleaning performance, enabling it to not only avoid the impact of outdoor moisture and sewage but also remove surface dust through natural rainwater (Figure 6b and Figure S26). To characterize the self‐cleaning performance of TCIP, a mud pouring experiment was conducted, with the non‐hydrophobic TCIP used as the control sample. During the mud pouring process, mud on the hydrophobic TCIP surface could slide off completely without residue (Figure 6c and Movie S3). In contrast, non‐hydrophobic TCIP led to substantial mud residue, which severely impaired its surface optical properties. Their reflectance spectra and digital photographs under cooling mode and heating mode were shown in Figure 6d and e, respectively. There was no mud residue on the surface of the hydrophobic TCIP, while a large amount of mud remained on the surface of the non‐hydrophobic TCIP. This severely affected the surface optical properties, especially in the cooling mode. As a result, the hydrophobic TCIP still maintained distinct dual‐mode reflectance after mud pouring and rinsing, whereas the non‐hydrophobic TCIP lost its radiative cooling capability. To determine the impact of mud residue on the surface temperature of samples, a simulated solar irradiation experiment was conducted indoors (Figure 6f). The indoor temperature was maintained at 23 ∼ 24°C. Under 1000 W/m^2^ solar irradiance for 20 minutes, the temperature of hydrophobic TCIP remained at 29°C, consistent with the transition temperature of 28°C and comfortable for the human body. However, the temperature of non‐hydrophobic TCIP reached 34°C, as the surface mud exhibited a certain photothermal effect, which obviously caused human discomfort.

When self‐adaptive materials are applied to building thermal management, their flame retardancy needs to be taken into consideration due to potential fire hazards [60]. Polymeric materials are widely used as the matrix for radiative cooling materials due to their easy processing and inherently high emissivity [61]. However, most polymers are flammable [62]. Unlike conventional polymer‐based radiative cooling materials, TCIP possesses high flame retardancy, which is endowed by its inorganic HNWs framework. To characterize the flame retardancy of TCIP, vertical burning tests were conducted, with commercial PVC wallpaper and cellulose paper as control samples (Figure S27). PVC and cellulose paper were completely burned within seconds when they contacted the flame, while TCIP retained nearly its original shape. This is attributed to the non‐flammability of the inorganic bundle network formed by the interwoven HNWs bundles. The TG curves of TCIP, TCMs, and HNWs were shown in Figure 6g. Due to their non‐flammability, HNWs retained 92% of their initial mass at 1000°C, with only a slight decrease around 300 °C, possibly caused by the pyrolysis of residual oleic acid and evaporation of moisture. TCMs were completely pyrolyzed during heating, as their organic composition is not heat‐resistant, with a thermal decomposition temperature of 308°C. TCIP exhibited a final residual mass of 68%, and its mass loss trend was consistent with that of TCMs, resulting from the decomposition of TCMs. This indicates that the HNWs, as the framework, were barely affected during heating, and the multi‐layered HNWs bundle network could serve as a protective layer against flames. The cross‐sectional SEM image of TCIP after flame exposure showed that its original multi‐layered structure was retained (Figure 6h). The reflectance spectrum of TCIP after exposure to flame showed that it still maintained high reflectivity, though slightly lower than that before combustion (Figure 6i). This may be attributed to the light absorption of the ash formed by TCMs. Additionally, TCIP retained a high mechanical performance after combustion, and could support a weight of 50 g without being damaged. Owing to its high flame retardancy, TCIP can be used as a fire‐resistant layer for buildings. We conducted a test with it serving as a barrier. As shown in Figure 6j and Movie S4, a model house was placed behind the protective cover made of TCIP. A butane torch capable of reaching a temperature of 1300°C was used as the fire source. Within 3 seconds of initial flame exposure, flames appeared on the TCIP surface due to the presence of organic TCMs. The appearance of flames is primarily attributed to the combustible nature of TCMs, whose uniform distribution within the inorganic HNWs network imparts brief combustibility to TCIP. This observation is consistent with the limiting oxygen index value of 23.5% measured for TCIP (Figure S28). The flames persisted for only 2 seconds and self‐extinguished once the TCMs were converted to ash. At this stage, the inorganic HNWs framework remained structurally intact and served as an effective barrier against further flame propagation. The model house remained intact throughout the experiment, as TCIP effectively blocked the flame. TCIP exhibits excellent flame retardancy and can be used as a high‐performance building surface material to eliminate potential fire hazards.

For building applications, long‐term optical stability and surface wetting durability are critical performance metrics. To rigorously evaluate these aspects, TCIP was subjected to accelerated aging tests for a total duration of 360 h. The test employed a long‐arc xenon lamp as the light source. A constant temperature of 60°C and a relative humidity of 60% were maintained, thereby subjecting the sample to a hot and humid environment. The optical performance of TCIP during the aging process is presented in Figure S29. Prolonged photoaging exerted a certain influence on the reflectance properties. Specifically, the reflectance in the cooling mode decreased from 94.1% to 92.0%, while the reflectance in the heating mode increased from 63.7% to 74.0%. The cooling mode reflectance exhibited only a slight decrease of 2.1%, whereas the heating mode reflectance showed a notable increase of 10.3%. These results indicate that extended photoaging has a minimal impact on the cooling mode reflectance but induces a distinct change in the heating mode reflectance. As revealed in the SEM images shown in Figure S30, after 360 h of photoaging, the bundled structure of the HNWs remained intact and undamaged, whereas a small number of fractures were observed in the TCMs. The preserved structural integrity of the HNWs bundles accounts for the sustained high reflectance in the cooling mode. Conversely, the occurrence of fractures within the TCMs is likely responsible for the observed increase in the heating mode reflectance. Although the optical performance of TCIP experienced a slight decline following prolonged photoaging, it still maintained a cooling mode reflectance of 92.0% and a spectral modulation capability of 20.1%. Furthermore, the hydrophobic properties after photoaging were also evaluated. The water contact angle of TCIP decreased only marginally from 146.8° to 140.8°, indicating that the material retained a high degree of hydrophobicity (Figure S31). This finding demonstrates that photoaging did not significantly compromise the hydrophobic performance of TCIP, which can be attributed to the PDMS component. The backbone of PDMS comprises Si─O bonds with high bond energy, endowing it with excellent resistance to ultraviolet radiation [63].

### 2.5 Building Energy Consumption Simulation of TCIP

**FIGURE 7:**
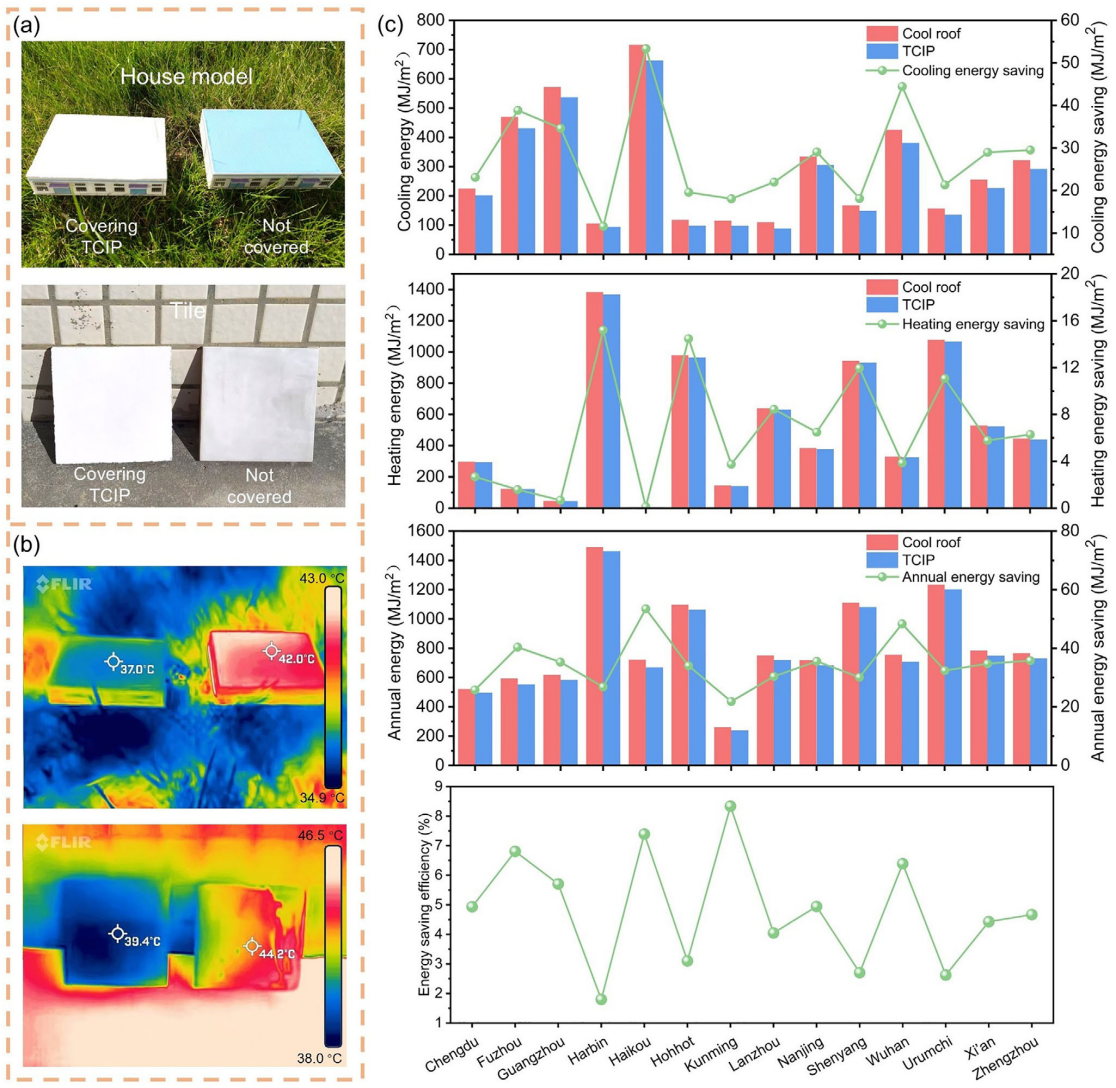
(a) Digital photographs of the house model and tile covered with TCIP. (b) The infrared thermographic images demonstrated a temperature decrease in both the house model and the tile after being covered with TCIP. (c) Simulations of the energy consumption of building in 14 typical cities in China. The comparisons were made between TCIP‐applied buildings and buildings with cold roof in terms of cooling energy consumption, heating energy consumption, total energy consumption, as well as the energy‐saving efficiency of TCIP.

TCIP achieves unique spectral properties through the selection of materials with specific optical properties and regulation of light propagation paths, enabling efficient switching between radiative cooling and solar heating. Employing the currently mature industrial papermaking process, large‐scale fabrication of TCIP can be facilely realized. Additionally, the hydrophobic self‐cleaning property and flame‐retardant property of TCIP ensure its stable operation in complex and variable external environments and eliminate potential fire safety hazards. In practical applications, TCIP can be applied to building envelopes for on‐demand building thermal management. To demonstrate its application, TCIP was applied to tiles and roof of house models (Figure 7a). In infrared thermal images, the house models and tiles covered with TCIP exhibited a significant cooling effect compared with the original blank samples, indicating their potential in thermal management (Figure 7b). To visualize the energy‐saving effect of TCIP, EnergyPlus (version: 23.2) was used to simulate the annual cooling, heating, and total energy consumption of two buildings: one with a TCIP‐covered roof and the other with a cool roof [64] (Figure 7c, Note S5 and Figure S32). Fourteen cities evenly distributed across different regions of China were selected as simulation objects. Due to its excellent radiative cooling performance, TCIP exhibited lower cooling energy consumption than cool roof. Notably, thanks to its self‐switching property, TCIP also had lower heating energy consumption than cool roofs, achieving energy savings in both cooling and heating. In terms of annual energy consumption, it can realize a maximum energy saving of 53 MJ/m^2^, with an energy‐saving efficiency of up to 8.3% compared to cool roofs. The above results indicate that TCIP has broad prospects in building thermal management, and its simple preparation process, self‐cleaning, flame‐retardant and high scalability properties provide an ideal option for building energy conservation.

## 3 Conclusions

In summary, TCIP with a multi‐layered bead‐network structure was developed via a layer‐by‐layer embedding strategy. Self‐assembled nano‐submicron HNW bundles and micron‐scale TCMs were selected as matrix materials. By leveraging the specific optical properties of the selected materials and utilizing the designed multi‐scale bead‐network structure, high cooling performance (R_cooling mode_ = 94.1%, E_ATW_ = 95.5%) and high spectral regulation capability (ΔR = 30.4%) were achieved. Through experiments and simulations, the regulation of light propagation paths in TCIP was systematically investigated, which provides a scientific reference for the design of advanced adaptive thermal management materials. Meanwhile, TCIP possesses both flame‐retardant and hydrophobic properties, and its high scalability endows it with advantages in practical applications to meet different application scenarios.

## 4 Experimental Section

### 4.1 Materials

Oleic acid, sodium dihydrogen phosphate, methanol, sodium hydroxide, and ethyl acetate were acquired from Shanghai Aladdin Biotech Co., Ltd. Calcium chloride and ethanol were sourced from Sinopharm Chemical Reagent Co., Ltd., and thermochromic microcapsules (TCMs, transition temperature: 28 °C) were provided by Shenzhen Huancai Color Changing Technology Co., Ltd. Polydimethylsiloxane (PDMS) prepolymer and its corresponding curing agent were purchased from the Dow Chemical Company. Additionally, the UV absorber [2‐(2′‐hydroxy‐5′‐tert‐cyclohexylphenyl)benzotriazole] and light stabilizer [bis(2,2,6,6‐tetramethyl‐4‐pyridyl) sebacate] were obtained from Dongguan Shanyi Plastic Technology Co., Ltd. All reagents employed in this study were used as received without additional purification.

### 4.2 Preparation of Hydroxyapatite Nanowires (HNWs)

HNWs were fabricated via a solvothermal approach, with calcium oleate serving as the precursor. Initially, 93.6 g of oleic acid and 47.5 g of methanol were dissolved in 135.0 mL of deionized water, followed by a 30‐minute stirring process. Next, 10.5 g of sodium hydroxide was dissolved in 150.0 mL of deionized water to form a NaOH solution; this solution was slowly added to the aforementioned mixture, and mechanical stirring was continued for another 30 minutes. In the meantime, 3.33 g of calcium chloride and 9.36 g of disodium hydrogen phosphate dihydrate were separately dissolved in 120 mL and 180 mL of deionized water, and the two solutions were then added to the mixed system. The resulting solution was transferred into a polytetrafluoroethylene‐lined stainless‐steel autoclave, which was sealed and heated at 180°C for 24 hours. After the reaction was completed, the obtained slurry was rinsed three times alternately with deionized water and ethanol. Finally, the HNWs were dispersed in water for subsequent use.

### 4.3 Fabrication of Thermochromic Inorganic Paper (TCIP)

TCIP was prepared via a mature pulping‐papermaking process. First, 50 mg of TCMs, 25 mg of UV absorbers, and 25 mg of light stabilizers were dispersed in 5 mL of absolute ethanol, mechanically stirred at room temperature for 1 h, and then vacuum‐filtered. The mixture was washed three times with deionized water and dried to obtain UV‐stabilizer‐modified TCMs, which were used to enhance the UV resistance of TCMs. The resulting TCMs were mixed uniformly with 250 mg of HNWs and dispersed in deionized water to prepare thermochromic inorganic pulp. Finally, the thermochromic inorganic pulp was processed by vacuum filtration and drying (80°C, 0.9 MPa, 10 min) in a rapid paper machine (95 854, Frank‐PTI) to obtain TCIP.

### 4.4 Hydrophobic Modification of Thermochromic Inorganic Paper

TCIP was immersed in a polydimethylsiloxane (PDMS) reaction solution (mass ratio of ethyl acetate: PDMS prepolymer: curing agent = 100:10:1) for 30 min and then taken out. The PDMS‐modified TCIP was heat‐cured in an oven at 100°C for 2 h to obtain hydrophobic TCIP.

### 4.5 Characterization

The microstructure and composition of the samples were obtained using a scanning electron microscope (Sigma 300, Zeiss) and a transmission electron microscope (Talos F200X, FEI). A Fourier transform infrared spectrometer (Nicolet iS10, ThermoFisher) was used to characterize the chemical groups and compositions of the samples. A differential scanning calorimeter (DSC 200 F3, Netzsch) was employed to test the endothermic and exothermic peaks of the samples. A universal mechanical tester (CMT6203, SZsansi) was used to measure the tensile strength of the samples. The refractive index and extinction coefficient of the samples were characterized using an ellipsometer (V‐VASE, J.A. Woollam). The size distribution of the samples was obtained via a nanoparticle size and Zeta potential analyzer (Zetasizer Nano ZS90, Malvern). A UV‐visible‐near infrared spectrophotometer (Lambda 750, PerkinElmer) was used to acquire the reflectance and transmittance spectra of the samples, and a Fourier transform infrared spectrometer (Nicolet iS50, ThermoFisher) was employed to obtain the emissivity spectra of the samples. The water contact angle of the samples was obtained using a contact angle goniometer (HK‐SPCA‐1, Harke). The pyrolysis curves of the samples were acquired via a thermogravimetric analyzer (STA 449 F3, Netzsch). The weathering resistance of the samples was evaluated using a xenon lamp weathering test chamber (SN‐500, Wxboleda). The tests were conducted with a long‐arc xenon lamp as the light source, operating at an irradiance of 550 ± 5 W/m^2^ over a spectral wavelength range of 290–800 nm. The chamber temperature was maintained at 60°C, and the relative humidity was set to 60%.

## Conflicts of Interest

The authors declare no conflicts of interest.

## Supporting information




**Supporting File 1**: advs75716‐sup‐0001‐SuppMat.docx.

**Supporting File 2**: advs75716‐sup‐0002‐Movie S1.mp4.

**Supporting File 3**: advs75716‐sup‐0003‐Movie S2.mp4.

**Supporting File 4**: advs75716‐sup‐0004‐Movie S3.mp4.

**Supporting File 5**: advs75716‐sup‐0005‐Movie S4.mp4.

## Data Availability

The data that support the findings of this study are available from the corresponding author upon reasonable request.
