# Supplementary material for: Self‐Adaptive Non‐Flammable Wallpaper With Layered Bead‐Network Structure for Light Path Modulation Enabling on‐Demand Building Thermal Management
Source: Adv Sci (Weinh). 2026 May 19;13(47):e75716. doi: 10.1002/advs.75716 (PMC13335862; doi:10.1002/advs.75716)
Supplement: Supplementary file 1 [file ADVS-13-e75716-s004.docx]

Supporting Information

**Self-Adaptive Non-Flammable Wallpaper with Layered Bead-Network Structure for Light Path Modulation Enabling On-Demand Building Thermal Management**

*Jianyu Wu, Linmin Xia, Jiankun Wu, Yuying Yang, Wuhong Zhong, Jianguo Li, Yan Yu*, and Rilong Yang**

J. Wu, Y. Yang, J. Wu, W. Zhong, Prof. J. Li, Prof. Y. Yu, Dr. R. Yang

College of Material Engineering

Fujian Agriculture and Forestry University

Fuzhou 350108, China

National Forestry and Grassland Administration Key Laboratory of Plant Fiber Functional Materials

Fuzhou 350002, China

Dr. L. Xia

School of Food and Liquor Engineering

Sichuan University of Science and Engineering

Yibin 644000, China

E-mail: rilongyang@fafu.edu.cn

yuyan9812@fafu.edu.cn

**Note S1. Calculation of net cooling power**

The net cooling power (P_net_) is calculated using the following equation^[1-3]^:

$P_{net}=P_{rad}-P_{amb}-P_{sun}-P_{conv+cond}$	(S1)

where the components are defined as follows:

*P_rad_(T)* represents the thermal radiation power emitted by the sample, expressed as:

$P_{rad}(T)=\int d\Omega cos\theta\int I_{BB}\left( T,\lambda\right)E\left( \lambda,\theta\right)d\lambda$	(S2)

*P_amb_(T_amb_)* denotes the thermal radiation power absorbed by the sample from the ambient environment:

$P_{amb}(T_{amb})=\int d\Omega cos\theta\int I_{BB}\left( T_{amb},\lambda\right)E\left( \lambda,\theta\right)E_{amb}\left( \lambda,\theta\right)d\lambda$	(S3)

*P_sun_* is the solar radiation power absorbed by the sample:

$P_{sun}=(1-R_{solar})P_{solar}$	(S4)

*P_conv+cond_* represents the heat transfer power caused by convection and conduction:

$P_{conv+cond}=hc\left( T_{amb}-T \right)$	(S5)

Neglecting the directional dependence and wavelength dependence of emissivity, the average emissivity is used instead. After simplification:

*P_rad_(T)* can be expressed using the Stefan-Boltzmann law:

$P_{rad}(T)\approx\sigma E_{ATWc}T^{4}$	(S6)

Similarly, *P_amb_(T_amb_)* is simplified to:

$P_{amb}(T_{amb})\approx\sigma E_{ATW}E_{amb}{T_{amb}}^{4}$	(S7)

where *σ*, *E_ATW_*, *E_amb_*, *T*, and *T_amb_* represent the Stefan-Boltzmann constant, the emissivity of the radiative cooling material, the emissivity of the environment, the temperature of the radiative cooling material, and the ambient temperature, respectively.
Under ideal conditions, the following parameters are adopted in the calculation^[4]^: ambient emissivity *E*_amb_ set to 0.724, and solar irradiance *P*_solar_ set to 1000 W/m². Based on these, the net cooling power under different *R*_solar_ with a fixed *E*_ATW_ =1 (Figure 2a) and under different *E*_ATW_ with a fixed *R*_solar_=1 (Figure 2b) was calculated when *T*=*T*_amb_.

**Note S2. Simulative calculation of the scattering efficiency of HNWs bundles**

To theoretically investigate the scattering properties of nanowires with different diameters, optical simulations were performed using the finite-difference time-domain (FDTD) method built in Ansys Lumerical software. For plane wave incidence, a two-dimensional model was adopted (Figure S12) to calculate the scattering cross sections (σ_scat_) of HNWs bundles with different diameters within the solar wavelength range (0.25–2.5 μm).

The geometric cross-section of the nanowire bundle is defined as:

σ_geom_=πr²	(S8)

Where r is the radius of the nanowire bundle.

The scattering efficiency is calculated as:

*Q_scat_* = σ_scat_/σ_geom_	(S9)

In the analysis, the diameter range of HNWs bundles was set to 0.2 μm–1.2 μm based on the statistically obtained particle size data, and the refractive index of HNWs bundles was set to 1.4 according to the data measured by the ellipsometer.

**Note S3. Calculation methods for average solar reflectivity and emissivity**

The solar reflectivity (*R*_solar_) and atmospheric window emissivity (*E*_AW_) were computed using the following formulas^[5,6]^:

$$R_{solar}=\frac{\int\begin{aligned} 2.5\mu m \\ 0.3\mu m \end{aligned}I_{AM1.5}(\lambda)R(\lambda)d\lambda}{\int\begin{aligned} 2.5\mu m \\ 0.3\mu m \end{aligned}I_{AM1.5}(\lambda)d\lambda} (S10)$$

and

$$E_{ATW}=\frac{\int\begin{aligned} 13\mu m \\ 8\mu m \end{aligned}I_{BB}(\lambda,T)E(\lambda)d\lambda}{\int\begin{aligned} 13\mu m \\ 8\mu m \end{aligned}I_{BB}\left（ \lambda,T \right）d\lambda} (S11)$$

*I*_AM1.5_(*λ*) represents the spectral irradiance of the AM 1.5 global solar spectrum, where *λ* denotes the wavelength, *R*(*λ*) is the solar reflectivity of the sample surface, *E*(*λ*) stands for the emissivity of the sample surface, $I_{BB}\left( \lambda,T \right)=\frac{2hc^{2}}{\lambda^{5}}\frac{1}{e^{hc/(\lambda k_{B}T)}-1}$ is the spectral radiance of a blackbody at temperature *T* (set to 298 K), h, c, and k_B_ denote Planck’s constant, the speed of light in vacuum, and the Boltzmann constant, respectively.

**Note S4. Monte Carlo photon transport simulation: comparison between hierarchical distribution and top-aggregated distribution**

A Monte Carlo photon transport simulation was implemented using Python. The simulation employed a plane-parallel slab model that is infinite in the lateral (x, y) directions and has a finite thickness in the z-direction. The slab thickness was set to 0.5 mm. The medium was uniformly divided into 20 layers along the thickness direction, with layer indices starting from 0 (layer 0 representing the top layer). A total of 50,000 photons were launched vertically from the top surface, with initial propagation direction pointing toward the bottom. The propagation process of each photon was simulated to calculate the probabilities of absorption by HNWs and TCMs, as well as escape from the top or bottom boundaries.

The hierarchical distribution and top-aggregated distribution were defined as follows:

Hierarchical distribution：TCMs are uniformly distributed across all layers, with the local volume fraction *f(i)* in each layer equal to the global volume fraction *f*：

$$f\left( i \right)=f$$

$$i=0,1,\ldots,N-1$$

Top-aggregated distribution: TCMs preferentially populate the top layers, filling layer by layer downward, while satisfying total mass conservation：

$$f\left( i \right)=\left\{ \begin{matrix} 1, & 0\leq i<m \\ f_{\text{last}}, & i=m \\ 0, & i>m \end{matrix} \right.$$

Here:$N$ is the total number of layers ($N=20$), $m=\lfloor N\times f\rfloor$ (the number of completely filled layers), and $f_{\text{last}}=N\times f-m$ (the remainder, $0\leq f_{\text{last}}<1$). If $f_{\text{last}}=0$, the partially filled layer does not exist (the TCMs volume fraction in layer $i=m$ is zero).

Mass conservation：

$$\sum_{i=0}^{N-1} f(i)=m+f_{\text{last}}=N\cdot f$$

That is, the total TCMs content is strictly equal between the hierarchical distribution and the top-aggregated distribution.

The parameter settings for HNWs and TCMs are as shown in **Table S1**.

**Table S1.** Parameters used in Monte Carlo photon transport simulations.

| **Parameters** | **Value** |
| --- | --- |
| HNWs scattering coefficient | 4, 6, 8, 10, 12 mm^-1^ |
| HNWs absorption coefficient | 0.02 mm⁻¹ |
| TCMs scattering coefficient | 3.0 mm⁻¹ |
| TCMs absorption coefficient (white state) | 1.0 mm⁻¹ |
| TCMs absorption coefficient (black state) | 20.0 mm⁻¹ |
| TCMs volume fraction | 5, 10, 15, 20, 25, 30 % |

**Note S5. Building energy consumption simulation with EnergyPlus.**

Building energy consumption simulations were conducted using EnergyPlus (version: 23.2) software, which calculated the cooling energy consumption, heating energy consumption, and total energy consumption from January 1 to December 31 for two types of buildings: one with TCIP-covered roof and the other with cool roof. The building model used for simulation consisted of two adjacent rooms, each with dimensions of 6m × 4m × 3m (**Figure S32**), and the roof area of the building is 48 m². The HVAC (heating, ventilation, and air conditioning) system was set to be activated for cooling when the indoor temperature exceeded 26 ℃ and for heating when it dropped below 18 ℃. Fourteen cities distributed across various regions of China were selected, including Chengdu, Fuzhou, Guangzhou, Harbin, Haikou, Hohhot, Kunming, Lanzhou, Nanjing, Shenyang, Wuhan, Urumchi, Xi’an and Zhengzhou. The weather data for these cities were obtained from the official EnergyPlus weather database ([energyplus.net/weather](https://energyplus.net/weather)).

The specified parameters of walls, windows, and roofs are as follows:

Wall: reflectivity=0.3, emissivity=0.9, heat transfer coefficient=0.446 W/(m^2^・K)

Window: solar heat gain coefficient=0.6, heat transfer coefficient=2.6 W/(m^2^・K)

TCIP roof in the cooling mode: reflectivity=0.941, emissivity=0.955, heat transfer coefficient=0.593 W/(m^2^・K). TCIP roof in the heating mode: reflectivity=0.637, emissivity=0.955, heat transfer coefficient=0.593 W/(m^2^・K)

Cool roof^[7]^: reflectivity=0.7, emissivity=0.9, heat transfer coefficient=0.593 W/(m^2^・K)

The temperature-dependent switching of solar reflectivity was implemented using the Energy Management System (EMS), an advanced feature in EnergyPlus. EnergyPlus provides a simple programming language, the Energy Plus Runtime Language, allowing users to write EMS programs that override the default control logic to achieve custom control strategies. In this study, a sensor was set up to output the temperature (T) of the roof material, and an actuator (actuator1) was configured with two preset states (cooling mode and heating mode). The following EMS program was written to switch the actuator state based on the temperature:

if T>28

set actuator1=cooling mode

else

set actuator1=heating mode

endif

**Figure S1.** (a) The reflectivity spectra and color residue of TCMs. (b) Digital photograph showing the weak bonding force between TCMs. (c) Digital photograph showing good bonding between TCMs and HNWs bundles.

**Figure S2.** (a-c) SEM images of the top surface of HNWs paper.

**Figure S3.** (a-b) SEM images of cross section of the HNWs paper.

**Figure S4.** The preparation process of TCIP.

**Figure S5.** (a) The tensile strength of TCIP. (b) Digital photos showing that TCIP can withstand a mass of 3200 times its own weight.

**Figure S6.** The tensile strength and reflectivity of TCIP doped with 20% nanocellulose: (a) Tensile strength and (b) reflectivity.

**Figure S7.** Si element mapping images of TCIP in EDS.

**Figure S8.** DSC curve of TCMs.

**Figure S9.** Digital image of HNWs slurry and HNWs pulp.

**Figure S10.** The different light-scattering abilities of HNWs before and after self-assembly.

**Figure S11.** Refractive index and extinction coefficient of HNWs bundles.

**Figure S12.** The two-dimensional model constructed in the FDTD simulation.

**Figure S13.** Transmittance of HNWs paper with different solid contents.

**Figure S14**. (a and b) The reflectivity spectra of hierarchical distribution in two modes: (a) Cooling mode and (b) Heating mode. (c and d) The reflectivity spectra of top-aggregated distribution in two modes: (c) Cooling mode and (d) Heating mode.

**Figure S15.** (a) The SEM image of TCMs. (b) The diameter distribution of TCMs.

| **R_Cooling mode_**  **(%)** | **R_Heating mode_**  **(%)** | **Spectral regulation capability (%)** | **Year** | **Reference number in manuscript** |
| --- | --- | --- | --- | --- |
| 92.3 | 78.9 | 13.4 | 2024 | [30] |
| 91.0 | 81.0 | 10.0 | 2024 | [32] |
| 94.7 | 87.9 | 6.8 | 2022 | [42] |
| 95.5 | 87.4 | 8.1 | 2025 | [43] |
| 86.9 | 68.9 | 18.0 | 2024 | [44] |
| 91.3 | 72.7 | 18.5 | 2022 | [45] |
| 91.0 | 72.8 | 18.2 | 2024 | [46] |
| 65.3 | 46.1 | 19.2 | 2024 | [47] |
| 88.1 | 63.5 | 24.6 | 2024 | [48] |
| 85.0 | 60.0 | 25.0 | 2023 | [49] |
| 50.0 | 19.0 | 31.0 | 2025 | [50] |
| 49.2 | 16.9 | 32.2 | 2024 | [51] |
| 90.0 | 57.0 | 33.0 | 2025 | [52] |
| 71.0 | 34.0 | 37.0 | 2024 | [53] |
| 94.1 | 63.7 | 30.4 | 2025 | This work |

**Table S2.** Comparison of cooling-mode reflectivity and spectral regulation capability of TCIP with other TCM-based thermal management materials.

**Figure S16.** The reflectivity spectra of PVC wallpaper and cellulose paper.

**Figure S17.** Relative humidity during the winter temperature tests.

**Figure S18.** Water contact angle of TCIP with different content of TCMs: (a) 0 mg, (b) 25 mg, (c) 50 mg and (d) 100 mg.

**Figure S19.** Digital photos illustrating that water droplets do not adhere to the TCIP surface.

**Figure S20.** Digital photos demonstrating that water droplets can roll on the TCIP surface.

**Figure S21.** (a-b) Water contact angles of TCIP after tape peeling and sanding tests: (a) Tape peeling. (b) Sanding.

**Table S3.** Comparison of TCIP without PDMS and TCIP.

|  | **TCIP without PDMS** | **TCIP** | **Change** |
| --- | --- | --- | --- |
| Weight | 0.295 mg | 0.348 mg | 0.053 mg |
| Water contact angle | 55.2° | 146.8° | 91.6° |
| Speed of mode change | ＜2s | ＜2s | No change |
| Transition temperature | 27.8 ℃ | 28.5 ℃ | 0.7 ℃ |
| Cooling mode reflectivity | 92.9% | 94.1% | 1.2% |
| Heating mode reflectivity | 65.8% | 63.7% | -2.1% |
| Spectral regulation capability | 27.1% | 30.4% | 3.3% |
| Limit oxygen index | 25.3% | 23.5% | -1.8% |

**Figure S22.** Water contact angle of TCIP without PDMS.

**Figure S23.** Demonstration of mode conversion speed of TCIP without PDMS and TCIP.

**Figure S24.** DSC curve of TCIP without PDMS.

**Figure S25.** The reflectivity of TCIP without and TCIP.

**Figure S26.** Digital photos demonstrating that water flow can carry away dust on the TCIP surface.

**Figure S27.** The vertical burning tests of PVC wallpaper, cellulose paper and TCIP.

**Figure S28.** The limit oxygen index of TCIP without PDMS and TCIP.

**Figure S29.** The cooling mode and heating mode reflectivity spectra after accelerate aging test: (a) cooling mode and (b) heating mode.

**Figure S30.** The SEM images of TCIP after accelerate aging test.

**Figure S31.** The water contact angle of TCIP after accelerate aging test.

**Figure S32.** Schematic diagram of two 6 m × 4 m × 3 m building models for EnergyPlus simulation.

References

[1]	J. Li, X. Wang, D. Liang, N. Xu, B. Zhu, W. Li, P. Yao, Y. Jiang, X. Min, Z. Huang, S. Zhu, S. Fan, J. Zhu, A Tandem Radiative/Evaporative Cooler for Weather-Insensitive and High-Performance Daytime Passive Cooling, *Science Advances* 8 (2022): eabq0411.

[2]	J. Mandal, Y. Fu, A. Overvig, M. Jia, K. Sun, N. Shi, H. Zhou, X. Xiao, N. Yu, Y. Yang, Hierarchically Porous Polymer Coatings for Highly Efficient Passive Daytime Radiative Cooling, *Science* 362 (2018): 315.

[3]	W. Li, S. Fan, Radiative Cooling: Harvesting the Coldness of the Universe, *Optics & Photonics News* 30 (2019): 32.

[4]	D. Zhao, A. Aili, Y. Zhai, S. Xu, G. Tan, X. Yin, R. Yang, Radiative Sky Cooling: Fundamental Principles, Materials, and Applications, *Applied Physics Reviews* 6 (2019): 021306.

[5]	R. Wu, C. Sui, T. Chen, Z. Zhou, Q. Li, G. Yan, Y. Han, J. Liang, P. Hung, E. Luo, D. Talapin, P. Hsu, Spectrally Engineered Textile for Radiative Cooling Against Urban Heat Islands, *Science* 384 (2024): 1203.

[6]	H. Zhong, Y. Li, P. Zhang, S. Gao, B. Liu, Y. Wang, T. Meng, Y. Zhou, H. Hou, C. Xue, Y. Zhao, Z. Wang, Hierarchically Hollow Microfibers as a Scalable and Effective Thermal Insulating Cooler for Buildings, *ACS Nano* 15 (2021): 10076.

[7]	E. Hosseini, E. Bou-Zeid, The Diverging Indoor, Outdoor, and Power Implications of Different Rooftop Photovoltaic System Designs, *Energy and Buildings.* 347 (2025): 116354.
